# Cohort profile: China undergraduate cohort for environmental health study

**DOI:** 10.1186/s12889-024-17915-1

**Published:** 2024-03-15

**Authors:** Peng Lu, Jiaming Miao, Liu Yang, Siqi Dou, Lei Yang, Chongjian Wang, Hao Xiang, Gongbo Chen, Tingting Ye, Lailai Yan, Shanshan Li, Yuming Guo

**Affiliations:** 1 School of Public Health, Binzhou Medical University, 346# Guanhai Rd, Shandong Yantai, China; 2 Department of Epidemiology and Statistics, School of Public Health, Hebei Medical University, Hebei Key Laboratory of Environment and Human Health, Shijiazhuang, China; 3 Department of Epidemiology and Biostatistics, College of Public Health, Zhengzhou University, Zhengzhou, Henan China; 4 Department of Global Health, School of Public Health, Wuhan University, 115 Donghu Road, Wuhan, Hubei China; 5 Department of Laboratorial Science and Technology, School of Public Health, Peking University, Beijing, China; 6 Air Quality Research Unit, School of Public Health and Preventive Medicine, Monash University, Level 2, 553 St Kilda Road, 3004 Melbourne, VIC Australia

**Keywords:** Environmental Pollution, Heavy metal, Metabolomics, Global climate, Cohort Profile

## Abstract

The China Undergraduate Cohort (CUC) is an ambispective cohort study with its major purpose to better understand the effects of lifetime environmental exposures on health outcomes. We recruited 5322 college students with an average age of 18.3 ± 0.7 years in China from August 23, 2019 to October 28, 2019. Follow-up surveys were conducted annually. The dataset comprises individual demographic data (e.g. age, sex, height, weight, birth date, race, home address, annual family income, contact information), health-related behavior data (smoking status, smoking cessation, passive smoking exposure, drinking habit, physical activity, dietary status), lifestyle data (physical exercise, dietary habit, length of time spent outdoors), disease history (respiratory disease history, cardiovascular disease history, urinary system disease history, etc.), mental health status data (sleep quality, self-reported stress, anxiety and depression symptoms), lung function and blood samples data. Preliminary results from our cohort have found the association between air pollution, summer heat and mercury exposure and lung function among young adults in China.

## Why was the cohort set up?

The importance of environmental pollution and global climate change on human health and well-being has become increasingly apparent, especially air pollution and global warming [1, 2]. Air pollution is one of the greatest impact factors on human health, and almost the entire global population (99%) breathes air that exceeds WHO air quality limits (https://www.who.int/). Air pollution killed an estimated 6.67 million people worldwide in 2019 [3]. Numerous studies have demonstrated that both long-term and short-term exposure to air pollution can cause various adverse impacts on human health, including premature death, respiratory diseases, cardiovascular diseases, and central nervous system dysfunctions [4–8]. Meanwhile, global warming has become one of the most serious global ecological crisis of the 21st century [9]. Global warming poses a comprehensive, multi-scale, and multi-level threat to global health. It not only affects ecosystems, economic activities, and human health but also has profound impacts on food production, water resource utilization, sea level rise, heatwaves, and the spread of infectious diseases. The harmful effects of global warming could exacerbate air pollution’s deleterious effects on human health [10].

With the unprecedented development of China’s economy, environmental pollution caused by heavy metal has become serious and widespread in most areas of China [11]. Heavy metals are collective term used for a group of metals having higher atomic number (above 20) and greater density (5 g/cm^3^) [12]. The most common toxic heavy metals included mercury, cadmium, selenium, etc. [13, 14]. These heavy metals are directly related with human health and biological toxicity problems [13]. Studies have shown that exposure to mercury induces gastrointestinal, neurotoxic, and nephrotoxic effects [15]. In addition, methylmercury causes mitochondrial damage, lipid peroxidation, accumulation of neurotoxic molecules, and disruption of microtubules [16]. Cadmium can cause serious health hazards such as kidney damage, prostate dysfunction, bone disease, and cancer [17, 18]. Furthermore, environmental heavy metals may alter the risks of diseases by interacting with psychological, nutritional, and physical activity factors.

The association between environmental exposures, behavioral factors and human health has been constantly reported [19–21]. However, the study that explored the directly, indirectly, and interactively impact of the exposure on human health is limited. In addition, the multiomics explanation of disease occurrence is scarce. To explore the possible health effects associated with short-term or long-term environment factors exposure and other socio-economical influencing factors, such as annual household income, the multiomic variation in disease development, and to provide evidence for disease prevention and health promotion in populations worldwide, the China Undergraduate Cohort (CUC) Study of Environment Exposure and Health was launched from 23 August 2019 based on standardized survey methods and rigorous quality control measures.

Large long-term cohort studies have huge potential to increase understanding of environmental exposures and disease and thus improve the health of current and future generations, but require sustained investment from funders and continued involvement by participants. Specific objectives of the CUC include:

- Investigating the effects of lifetime environmental exposures (e.g., indoor air pollution, outdoor air pollution, ambient temperature, residential green space, internal metal exposure, and nutrition) and lifestyle factors (smoking, drinking, physical activity) on molecular changes of health outcomes (metabolomics).
- Estimating the association between environmental or lifestyle factors and sub-clinical or clinical outcomes in young adulthood period including lung function, cardiovascular function and urologic function.
- Exploring the associations between environmental exposures and metabolomic changes or metabolomic signatures in blood.
- Exploring the associations between metabolomic changes and sub-clinical or clinical outcomes in young adult and in later life period.
- Exploring the multiomics linkage between environmental pollutant exposure and genomics, transcriptomics, proteomics and metabolomics, and the early omic changes of disease.

### Who is in the cohort?

**Fig. 1:**
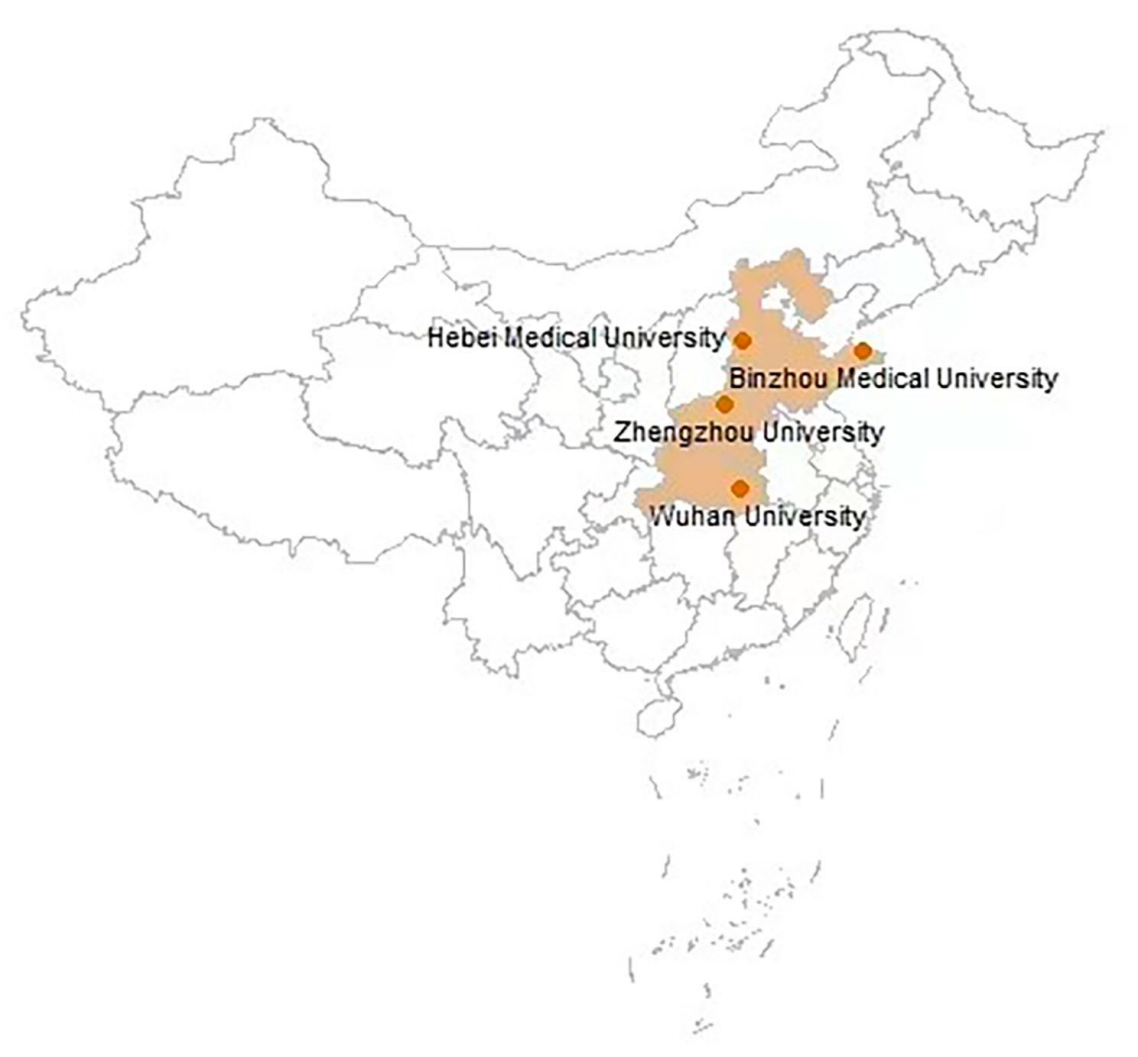
The locations of the four medical college

**Fig. 2:**
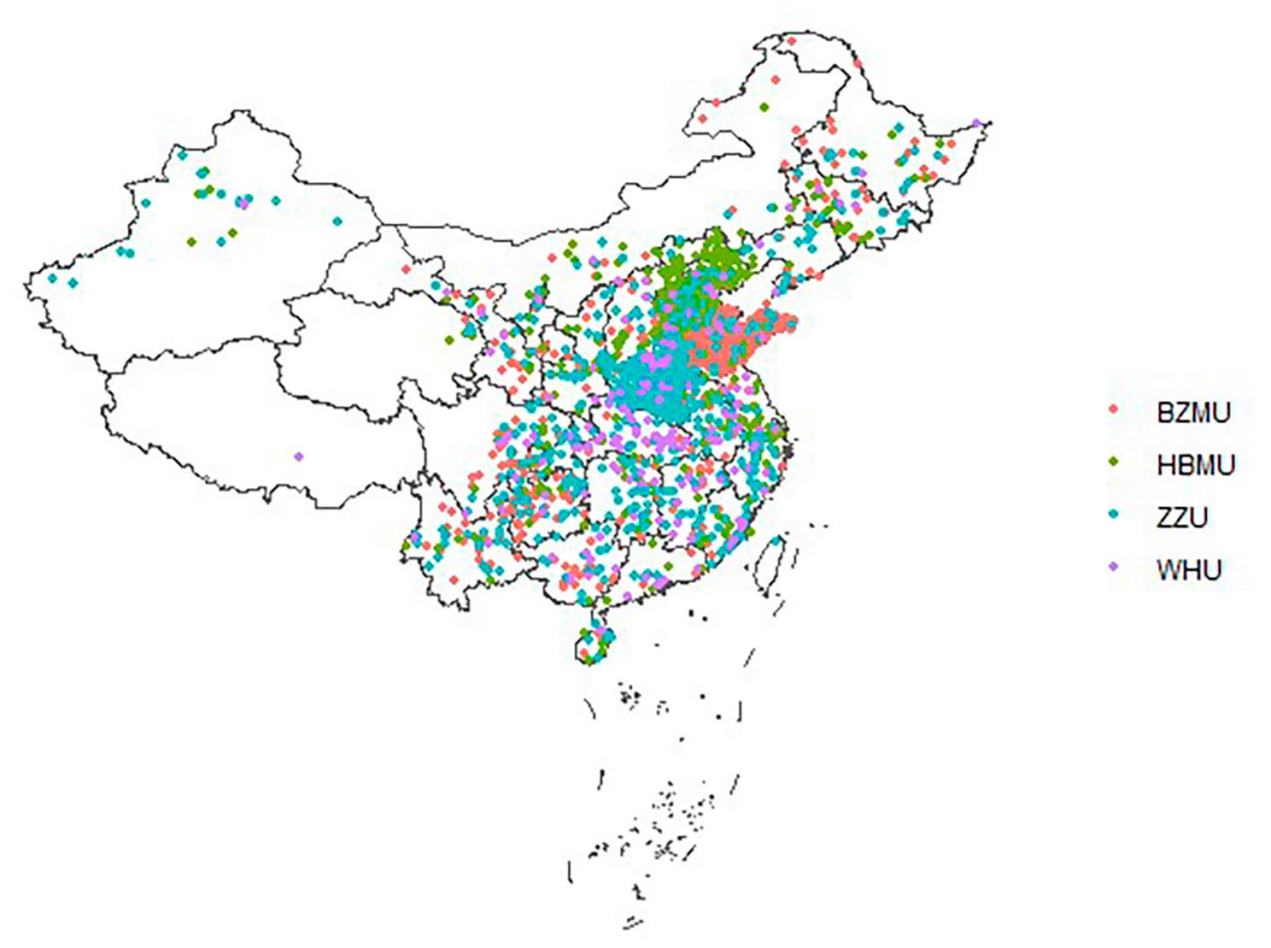
The detailed home location distribution of study participants Note: BZMU: Binzhou Medical University, HBMU: Hebei Medical University. ZZU: Zhengzhou University, WHU: Wuhan University

The CUC is an ambispective cohort study recruited 5322 undergraduates aged 18.3 ± 0.7 years at baseline, from across China. Participants were recruited in four universities, namely Binzhou Medical University (2733, 83.2% of the 2019 freshmen), Hebei Medical University (1130, 41.7% of the 2019 freshmen), Zhengzhou University (1126, 10% of the 2019 freshmen ), and Wuhan University (333, 7% of the 2019 freshmen ), from 23 August 2019 to 28 October 2019. The locations of these four universities are shown in Fig. 1. The detailed home location of the students is shown in Fig. 2.

We used the random sampling method to choose study subjects. Students enrolled in the four universities in 2019 were chosen as a cluster. Before carrying out this study, we distributed an informed consent form to each student, introducing the purpose of the study and the operations required: drawing venous blood, lung function measurement, questionnaire survey, etc., and promised to protect their private information, ensured their informed consent and finally obtained their signature. All our methods were performed in accordance with relevant guidelines and regulations, and the Ethics Committee of Binzhou Medical University approved the study.

The baseline data collection was conducted during 23 August 2019 and 28 October 2019, at the time of the compulsory college-entry health examinations. This included a self-reported questionnaire, lung function test, and blood sample collection. The first follow-up data collection was conducted during 16 April 2020 and 23 April 2020, almost three months after the implementation of mass quarantine.

The uniqueness of this cohort is that it combines molecular aspects and subclinical parameters of respiratory and mental diseases with the interplay of environmental exposures in young adulthood.

The baseline demographic and lifestyle characteristics of the CUC cohort participants are presented in Table 1. The cohort has a total of 5322 paricipants, This is a young adult cohort with a slight majority of women and a small proportion of smokers. There were 12.7% of participants who had suffered from respiratory disease, and 14.5% reported that their parents had a respiratory disease history. Approximately one-half of respondents’ socio-economic status was advantageous (annual family income ≥ 50000RMB).

**Table 1:** Population characteristics and lung function outcomes (*n* = 5322)

Participant characteristics	2019	2020
Demographic characteristic		
Age (years)	18.3 ± 0.7	19.3 ± 0.7
Female (%)	3285 (61.7)	3285 (61.7)
Height(cm)	167.9 ± 8.4	168.3 ± 8.4
Weight(kg)	63.0 ± 16.6	64.0 ± 18.1
Cigarette smoke exposure (%)		
Smoking status	35 (0.6)	39 (0.7)
Passive smoking	572 (10.7)	392 (7.4)
Alcohol consumption	187 (3.5)	194 (3.6)
Physical activity intensity (%)		
> once a week	3877 (72.8)	3834 (72.0)
Respiratory disease history (%)		
Yes	677 (12.7)	825 (15.5)
No	4645 (87.2)	4497 (84.5)
Family history of lung disease (%)		
Yes	772 (14.5)	772 (14.5)
No	4550 (85.5)	4550 (85.5)
Socioeconomic status (%)		
Socioeconomic-advantage	2661(50.0)	2661(50.0)
Socioeconomic-disadvantage	2661(50.0)	2661(50.0)

## How often will they be followed up?

Participants completed baseline questionnaire, blood sample collection, and lung function test during the recruitment period in 2019. We collected mobile phone numbers, WeChat and QQ (a social networking site) numbers at baseline. We have multiple contact routes to follow up with individuals. Participants will undertake a follow-up study, including a questionnaire, and lung function test annually. The follow-up study blood sample collection will be conducted at the time when they are about to leave Yantai forinternship.

**Fig. 3:**
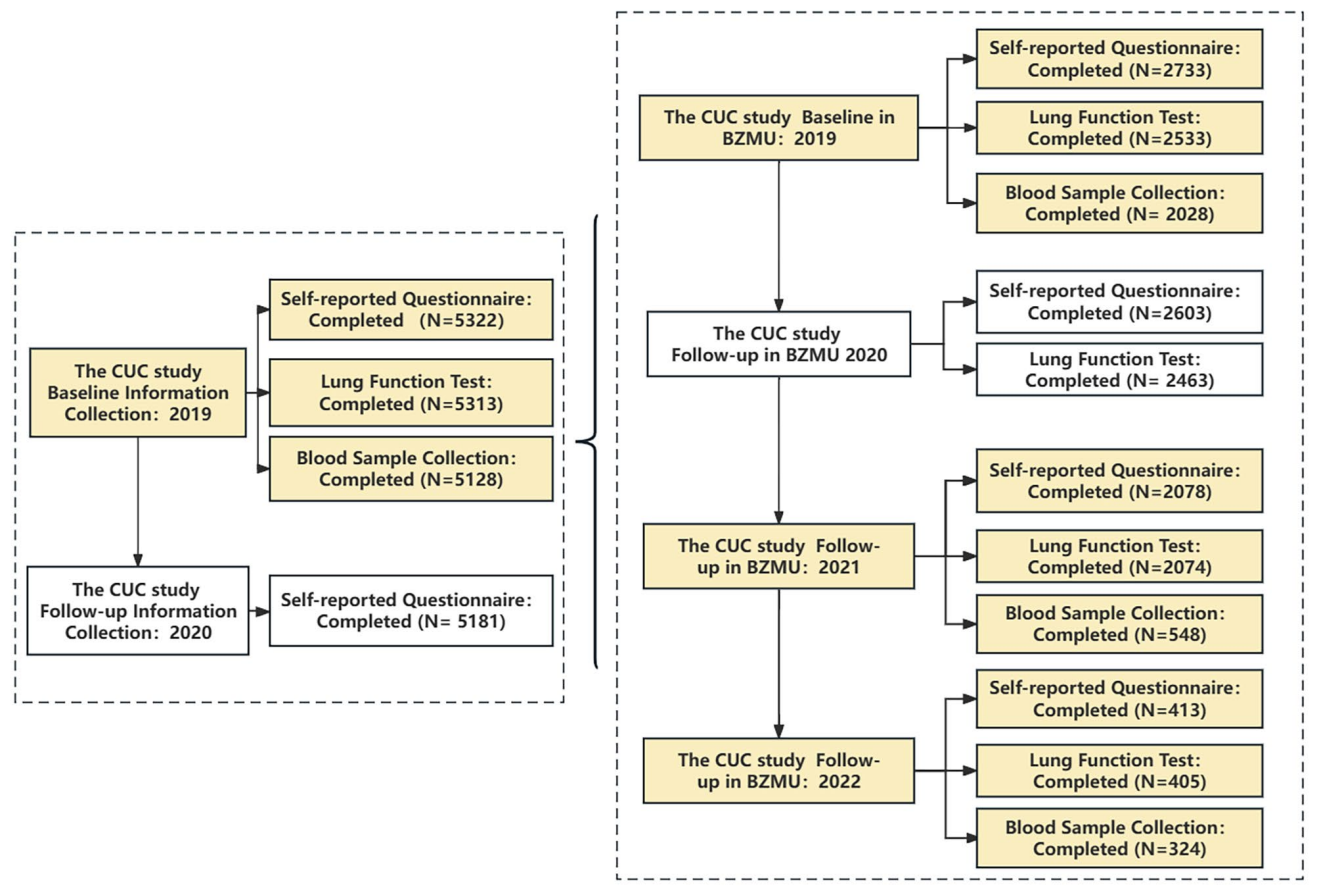
Study scheme of the CUC cohort Note: The yellow portion in the figure represents the blood samples collection in baseline and follow-up

Figure 3 shows the scheme of the CUC cohort study. The recruitment of the participants started in August 2019. Baseline data collection was conducted at four schools (namely, BZMU, HBMU, ZZU and WHU) from 23 August to 28 October, 2019. Subsequently, we conducted the first follow-up at four schools in April, 2020 to explore the impact of COVID-19 on the psychological changes of undergraduate students. Response rates of the first follow-up were high (97.35%). The right half of Fig. 3 shows the BZMU baseline and follow-up results. In 2019, we collected baseline data from the participants, which mainly included self-reported questionnaires, blood samples, and lung function data. In 2021 and 2022, we conducted follow-up visits in batches based on the participants’ internship schedules before leaving Yantai. For example, some of the participants who were about to leave Yantai for internship in 2021 were followed-up. There were 625 participants completed data collection in 2019 baseline (not shown in Fig. 3). There were 548 finished the follow-up data collection in 2021. The follow-up rate is 87.7%. The specific follow-up process is shown in Fig. 3.

## What has been measured?

The purpose of the CUC cohort study was to explore the association between lifetime environmental exposure (such as air pollution, ambient temperature, surrounding greenness) and behaviors (smoking, drinking, physical activity) on health outcomes (physical and mental health). Table 2 summarized the measurements collected from the CUC study, which consisted of a self-reported questionnaire, lung function test, fasting blood and exposure characterization. More details of these measurements are described below.

**Table 2:** Data collected from CUC study

Measurements	Variables
Electronic questionnaire	
Demographics and socio-economic status	Sex, ethnic group, date of birth, national ID number, contact information, height, weight, tablets use, household income, household population
Address information	Home address before college, high / junior high/primary school name and address^a^, home address at birth day^a^
Smoking	Current/past smoker, starting age smoking, number of cigarettes, passive smoking exposure
Alcohol consumption	Drinking status, types of drink, frequency of drinking
Home cooking	Cooking oil type, cooking fuel type, ventilation method during cooking, cooking method
Diet	Frequency of three meals a day^b^, staple food type, different foods consumed type, diet taste
Water and other beverages	Daily water intake, drinking water type, drinking water temperature, favorite beverage type, drinking other beverages frequency
Refrigeration	Whether to install cooling equipment^b^, whether use of air-conditioning^b^, air conditioner setting temperature^b^
Heating	Heating type^b^
Outdoor	Average daily outdoor stay time
Physical activity	Whether to exercise in the past three months
Sleep	Pittsburgh sleep quality index (PSQI)
Disease history and family history	
Ophthalmology diseases	Conjunctivitis, iritis, nearsightedness, farsightedness, other ophthalmology diseases
Respiratory diseases	Allergic rhinitis, asthma, chronic bronchitis, pneumonia, emphysema, tuberculosis, other respiratory diseases
Digestive system diseases	Gastrointestinal disease, liver disease, gallbladder disease, pancreatic disease, other digestive system diseases
Oral disease	Mouth ulcers, bleeding gums, caries, periodontitis, other oral disease.
Genitourinary system diseases	Nephritis, kidney stones, cystitis, gout, polycystic ovary, other genitourinary system diseases
Cardiovascular diseases	Hypertension, heart disease, hyperlipidemia, other cardiovascular diseases
Endocrine system diseases	Diabetes, thyroid disease, hashimoto’s thyroiditis, other endocrine system diseases
Mental diseases	Depression, anxiety, mania, schizophrenia, obsessive compulsive disorder, suicidal tendency, other mental diseases
Skin diseases	Eczema, dermatitis, vitiligo, psoriasis, systemic lupus erythematosus, other skin system diseases
Tumor	Leukemia, lung cancer, liver cancer, stomach cancer, osteosarcoma, other tumor
Nervous system disease	Nervous headache, dizziness, epilepsy, other nervous system disease
History of other diseases	
Treatment situation	Whether the above disease has been treated, hospitalization in the past three years, injuries and diagnosis and treatment in the past two weeks
Menstruation (for women)	Age of menarche, Menstrual cycle, menstrual duration, menstrual volume, dysmenorrhea, whether the menstrual cycle regular^c^, psychological feelings before and during menstruation^c^, Physical sensation before and during menstruation^c^
psychological conditions	7-item generalized anxiety disorder (GAD-7), patient health questionnaire depression (PHQ-9)
Takeout^c^	Frequency of takeout, favorite type of takeaway cooking, favorite type of takeaway food, takeaway lunch box
Medical examination	
Lung function	Slow vital capacity, forced vital capacity, maximum voluntary ventilation
Fasting blood	Heavy metal, Metabolomics
Exposure characterization	
Daily air pollution data	Sulfur dioxide, nitrogen dioxide, nitric oxide, particles with an aerodynamic diameter of 10 μm or less (PM_10_), particles with an aerodynamic diameter of 2.5 μm or less (PM_2.5_), ozone (China National Environmental Monitoring Centre)
10*10km daily average air pollution data	PM_2.5_, ozone (Tracking Air Pollution in China)
Daily meteorological data	Air pressure, air temperature, relative humidity, wind direction and wind speed (National Meteorological Information Centre)
Residential greenness	Normalized Difference Vegetation Index (NDVI) and Enhanced Vegetation Index (EVI)

Note: ^a^ Include this questions at baseline and fourth follow-up

^b^ The baseline included primary school, junior high school, and high school stages

^c^ Questions added during the fourth follow-up

## Self-reported questionnaire

The self-reported questionnaire was developed by professionals and supplemented or revised every year based on different study purposes v. During or after the lung function test, participants were asked to fill out the questionnaire online. The main content of the questionnaire includes demographics and socio-economic status (e.g. national ID number, age, sex, height, weight, birth date, race, home address, annual family income, contact information), health-related behavior data (smoking status, smoking cessation, passive smoking exposure, drinking habit, physical activity, dietary status), disease history (respiratory disease history, cardiovascular disease history, urinary system disease history), lifestyle data (physical exercise, dietary habit, length of time spent outdoors) and mental health status data (sleep quality, self-reported stress, anxiety and depression symptoms).

### Lung function test

We have conducted lung function test once a year since 2019 in BZMU. The lung function test was conducted indoor by Gest HI-101 spirometer (Chest, Tokyo, Japan) and performed according to the European Respiratory Society specifications [22]. A complete lung function test contains three projects: slow vital capacity (SVC), forced vital capacity (FVC), and maximum voluntary ventilation (MVV). Specific quality controls were performed as follows: (1) all tests were done by well-trained professionals; (2) spirometers were calibrated before daily lung function test or after replacing; (3) no blocked mouthpiece, air leakage, early termination or cut-off expiration of any detector; (4) extrapolation volume less than 150mL or 5% of the FVC, and a forced expiratory time exceeding 6s; (5) expiratory platform in volume-time curve and reproducible test with three acceptable flow-volume curves.

### Biomarkers of internal metal

During the baseline and follow-up, we used heparin anticoagulation tube to draw 10 ml fasting blood. The fasting blood samples were separated under strict control into 1 ml EP tubes, which was affixed with a freezing label with personal information. A total of 10 tubes were divided, including 1 tube of whole blood, 2–4 tubes of serum and 2–5 tubes of blood cells. After aliquot, we put it into the refrigerator at -80℃ for freezer storage. Part of the samples were transferred to the Peking University Experimental Center to test internal metal concentration and metabolomic signatures. In June 2021, we used Inductively Coupled Plasma Mass Spectrometry (ICP-MS) with dynamic reaction cell to determine the heavy metal content in fasting blood samples of the CUC participants in the Peking University Experimental Center. We took 0.25 ml of blood and made it into 8 ml of measurement solution through a series of procedures such as microwave digestion, and added indium as an internal standard [23]. All measurement procedures were carried out in accordance with standardized protocols.

### Exposure characterization

#### Ambient air pollution

Individual exposure to particulate matter ≤ 2.5 μm in diameter (PM_2.5_), particulate matter ≤ 10 μm in diameter (PM_10_), sulphur dioxide (SO_2_), nitrogen dioxide (NO_2_) and ozone (O_3_) were calculated at residence level. We obtained daily PM_2.5_, PM_10_, SO_2_, NO_2_ and O_3_ data from China National Environmental Monitoring Center (http://www.cnemc.cn/). Each participants’ home address was collected by questionnaire. We matched the latitude and longitude location of these addresses to the nearest monitoring station to estimate their air pollutant exposure information. In addition, we obtained 1 km×1 km maximum 8-hour O_3_ grid and PM_2.5_ data from the website Tracking Air Pollution in China (http://tapdata.org.cn/). This dataset was developed by a machine learning (ML) model, integrating the ground monitored air pollution data, satellite-derived aerosol optical depth (AOD), satellite auxiliary covariates, meteorological data, weather research and forecasting (WRF) and community multi-scale air quality (CMAQ) simulations. The PM_2.5_ five-fold cross-validation coefficients of determination 0.80–0.88, root-mean-square error 13.9–22.1 µg/m^3^, O_3_ five-fold cross-validation coefficients of determination 0.70, root-mean-square error 26 µg/m^3^. They are of high quality and has been widely used in previous studies on health impact assessment of air pollution [24–26].

#### Ambient temperature

We obtained data of daily mean temperature, relative humidity and wind speed from China Meteorological Data Sharing Service System (http://data.cma.gov.cn). The dataset provided by the system includes meteorological data from all weather stations in China and is free to users. We matched the latitude and longitude location of the participants’ home address and school address to the nearest meteorological station to estimate their ambient temperature exposure information.

#### Residential greenness

We calculated participant’s surrounding greenness within the buffer radiuses of 500 m, 1000 and 3000 m based on home and school address. Normalized Difference Vegetation Index (NDVI) and Enhanced Vegetation Index (EVI) were used to represent the surrounding greenness of participants. NDVI derived from remote-sensing images with high spatial-temporal resolution can better reflect the change of vegetation greenness [27]. EVI is an improvement of NDVI, which is superior to NDVI in reducing background and atmospheric effects and saturation issues [28]. Surrounding greenness data was obtained from Moderate Resolution Imaging Spectroradiometer (MODIS) NDVI and EVI of the MODIS/Terra Vegetation Indices 16-Day L3 Global 250 m SIN Grid (MOD13Q1) providing continuous measurements for every 16 days at a spatial resolution of 500 m [29].

### What has it found? Key findings from the baseline study

A summary of published articles within the framework of the CUC cohort is given in Table 3. Below we highlight findings from our studies focusing on environmental influences on health. Details of these findings are described below.

**Table 3:** The summary of published articles in the CUC cohort study

Title of article	Year
Mental health of new undergraduate students before and after COVID-19 in China [30]	2021
Long-Term Improvement of Air Quality Associated with Lung Function Benefits in Chinese Young Adults: A Quasi-experiment Cohort Study [31]	2022
Life-time Summer Heat Exposure and Lung Function in Young Adults: A Retrospective Cohort Study in Shandong China [32]	2022
Association between Mercury Exposure and Lung Function in Young Adults: A Prospective Cohort Study in Shandong, China [33]	2023

#### Air pollutants and lung function

During September 2019 and September 2020, we used the CUC cohort dataset to investigate whether the reduction of long-term air pollution was associated with the improvement of lung function in Chinese young adults. We found that air pollutants concentrations during COVID-19 quarantine period in 2020 decreased significantly in comparison with the same period in 2019. The study has shown that every 5 µg/m^3^ decrease in annual average PM_2.5_ concentrations was associated with 36.0 ml [95% confidence interval (CI):6.0, 66.0 ml], 46.1 ml (95% CI:16.7, 75.5 ml), and 124.2 ml/s (95% CI:69.5, 178.9 ml/s) increment in the FVC, forced expiratory volume in 1 s (FEV_1_) and forced expiratory flow at 50% of FVC (FEF_50_) respectively. Similar associations were found for PM_10_. Among all the outcome variables, the small airway index FEF_50_ was more sensitive to pollutants. After adjusting for gaseous pollutants, the PM impact was estimated to be stronger.

#### Ozone and lung function

We conducted a prospective cohort study among 1594 college students with a mean age of 19.2 years at baseline in Shandong, China from September 2020 to September 2021. Daily 10 km×10 km ozone concentrations come from a well validated machine learning approach and the time-weighted average concentrations during 12 months before the lung function test at the residential and school address was defined as the long-term ozone exposure. We found that each interquartile range (IQR) (8.9 µg/m^3^) increase in long-term ozone exposure were associated with a − 204.3 (95% CI: −361.6, − 47.0) ml/s, − 146.3 (95% CI: −264.1, − 28.4) ml/s, and − 132.8 (95% CI: −239.2, − 26.4) ml/s change in FEF_25_, FEF_50_, and FEF_25 − 75_, respectively. Stratified analyses found stronger adverse associations among female participants or those with a BMI ≥ 24 kg/m^2^.

#### Summer heat exposure and lung function

We performed a retrospective cohort study to investigate the association between lifetime heat exposure and lung function of young adults in 2020 [32]. We found that each 1 °C increase in life-time summer mean temperature was associated with 1.07% (95% CI: −1.95, − 0.18%) decrease in FVC and 0.88% (95%CI: −1.71, − 0.05%) decrease in FEV_1_. In the time window analysis, we found similar association in pre-school stage. For pre-school stage, each 1 °C increase in mean temperature was associated with 0.96% (95%CI: −1.69, − 0.22%) decrease in FVC and 0.82% (95%CI: −1.51, − 0.12%) decrease in FEV_1_. Stratified analysis showed that the lung function of the participants with respiratory disease history and non-cooling facility users were more likely to be affected by summer temperature. Fan and air conditioning using are the effective ways in reducing the deleterious effects of heat on lung function in summer [32].

#### Mass quarantine and mental health

We used participants of CUC to examine the change in mental health status and sleep quality of Chinese undergraduate students before and after COVID-19 mass quarantine. We found that compared with the baseline information in 2019, the mental health of undergraduate students was not deteriorated after the COVID-19 mass quarantine in 2020. During the quarantine period, the time spent on mobile gadgets increased and the time spent outdoors reduced significantly. Self-reported sleep quality improved after mass quarantine.

#### Heavy metals and lung function

In 2021, the CUC data were used to explore the association between blood heavy metal exposure and lung function. We explored the effects of mercury and nickel exposure on lung function separately. We found that both mercury and nickel exposure was associated with the lower FVC and FEV_1_ among Chinese young adults. In addition, we found that participants with high blood mercury concentrations, men and people who consumed fish more than once a week seem to be more sensitive in lung function changes. We also found that compared to participants with low nickel levels (≤ 25th percentile), those with intermediate (25-75th percentile) and high (> 75th percentile) nickel levels showed more decrease in FVC and FEV_1_. Furthermore, we found that the effect of nickel on lung function was stronger in urban participants than rural participants.

#### Heavy metals and mental health

We conducted a longitudinal cohort study to analyze the association between blood mercury levels and depression status based on the CUC cohort study in 2022. We found that for 2-fold increase in blood mercury levels, depressive symptoms score increased by 0.51 (95% CI: 0.15, 0.87). The association was sex-specific which affected men more than women. Similarly, the impact of mercury exposure was found stronger in those who consume fish at least once a month. In addition, blood mercury levels were more significantly associated with depressive symptoms scores in participants with lower household income, which may be due to their food and fuel choices.

### What are the main strengths and weaknesses?

This study has some limitation worth to mention. First, the questionnaires collected in this study contained a lot of self-reported information, which inevitably leads to certain recall biases, such as home and school addresses at various stages and lifestyle factors. Second, although our study participants came from all over China, the study participants only included undergraduates. The sample may not fully represent the entire population in China. Low education group is underrepresentation in our cohort. Third, due to the COVID-19 pandemic, we were only able to conduct follow-up visits to BZMU university and could not conduct the same follow-up study in other universities at the same time. Fourth, the cohort design has stronger causal inference than other observational design, weaker causal inference than randomized controlled trial.

However, as a prospective cohort study, we conducted repeated follow-ups, enabling us to explore the impact of short-term and long-term changes in environmental factors on health outcomes.The cohort design has stronger causal inference than other observational studies.Given the refined exposure information of CUC, it will allow the investigation of environmental effects upon health, even if effects are small. In addition, information for multiple environmental exposures will allow environment-environment interactions to be investigated. We also include a variety of health outcomes, such as lung function, mental health, heavy metal exposure levels and metabolomics, which allow us to explore the association between environmental factors and health as much as possible.

### Can I get hold of the data? Where can I find out more?

To maximize the use of the CUC data, we welcome new collaborations with other investigators. Currently, the database is not available for public download due to some sensitive information. Researchers interested in collaborating on studies related to the China Undergraduate Cohort study should contact the principal investigator, Peng Lu [peng.lu@monash.edu] and Yuming Guo [yuming.guo@monash.edu].

## Conclusions

The CUC established an ambispective cohort study that collected information on environmental exposures, intra-biological exposures and diverse health outcomes. CUC aimed to investigate the causal relationships between short-term or long-term exposure to environmental factors and other socioeconomic effects and health outcomes, to explore associations between environmental exposures and multiple omics, and to identify associations between metabolomic changes and subclinical or clinical outcomes. CUC provide evidence for disease prevention and health promotion in a global population.

## Data Availability

Data described in the manuscript, code book, and analytic code will be made available upon request pending application to and approval from the first author PL or the corresponding author YG.
